# Supplementary material for: A Machine Learning Model Based on PET/CT Radiomics and Clinical Characteristics Predicts ALK Rearrangement Status in Lung Adenocarcinoma
Source: Front Oncol. 2021 Mar 2;11:603882. doi: 10.3389/fonc.2021.603882 (PMC7962599; doi:10.3389/fonc.2021.603882)
Supplement: Supplementary file 1 [file DataSheet_1.docx]

**Supplementary methods**

The formulas of radscore calculation.

The formula for PET/CT radscore is:

Radscore= = 0.29*CT_ShortRunEmphasis_AllDirection_offset1_SD+0.142*CT_LongRunHighGreyLevelEmphasis_angle90_offset4+-0.769*CT_uniformity+-0.062*CT_HaralickCorrelation_AllDirection_offset4_SD+-0.055*CT_LongRunLowGreyLevelEmphasis_AllDirection_offset4_SD+-0.506*CT_HaraEntroy+0.802*CT_LongRunEmphasis_AllDirection_offset1_SD+-0.103*CT_Percentile70+-0.625*CT_LongRunEmphasis_AllDirection_offset4_SD+0.565*PET_Percentile10+-0.379*CT_GLCMEnergy_angle135_offset7+0.196*CT_LongRunLowGreyLevelEmphasis_AllDirection_offset1_SD+0.406*CT_LongRunHighGreyLevelEmphasis_angle0_offset1+0.432*CT_Percentile30+0.045*CT_LongRunEmphasis_angle135_offset4+-0.206*CT_LongRunLowGreyLevelEmphasis_AllDirection_offset7_SD+0.408*CT_GLCMEntropy_angle90_offset1+0.205*CT_HaralickCorrelation_AllDirection_offset7_SD+-0.361*CT_LongRunHighGreyLevelEmphasis_angle45_offset1+-0.132*CT_Correlation_AllDirection_offset4_SD+0.596*CT_LongRunEmphasis_angle90_offset4+0.751*PET_differenceEntropy + -0.757

The formula for CT radscore is:

Radscore= = 0.876*CT_ShortRunEmphasis_AllDirection_offset1_SD+0.021*CT_HaralickCorrelation_AllDirection_offset4_SD+-0.054*CT_Percentile65+-1.206*CT_HaraEntroy+0.432*CT_LongRunHighGreyLevelEmphasis_angle0_offset1+0.401*CT_LongRunLowGreyLevelEmphasis_AllDirection_offset1_SD+-0.167*CT_Correlation_AllDirection_offset4_SD+-0.437*CT_LongRunEmphasis_AllDirection_offset4_SD+-0.103*CT_Percentile10+-0.018*CT_GLCMEnergy_angle45_offset7+0.035*CT_ShortRunHighGreyLevelEmphasis_AllDirection_offset4_SD+-1.158*CT_GLCMEnergy_angle135_offset1+0.066*CT_Percentile85+0.278*CT_HaralickCorrelation_AllDirection_offset7_SD+0.496*CT_LongRunEmphasis_angle90_offset4+0.599*CT_Percentile35+0.233*CT_ShortRunEmphasis_AllDirection_offset1 + -0.599

The formula for PET radscore is:

Radscore =

2.002*PET_Percentile15+0.141*PET_differenceEntropy+-0.433*PET_Variance+-0.407*PET_HaralickCorrelation_angle0_offset1+-0.259*PET_MeanDeviation+-0.296*PET_ShortRunLowGreyLevelEmphasis_angle135_offset1+0.31*PET_LongRunHighGreyLevelEmphasis_angle90_offset4+0.146*PET_Elongation+0.029*PET_Percentile45+0.753*PET_Inertia_angle90_offset1+-0.955*PET_MinIntensity + -0.822
