# Supplementary material for: A Machine Learning Model Based on PET/CT Radiomics and Clinical Characteristics Predicts ALK Rearrangement Status in Lung Adenocarcinoma
Source: Front Oncol. 2021 Mar 2;11:603882. doi: 10.3389/fonc.2021.603882 (PMC7962599; doi:10.3389/fonc.2021.603882)
Supplement: Supplementary file 2 [file DataSheet_2.doc]

**Supplementary Figures legends**

**Supplementary Figure S1** Construction of a CT radiomic model based on CT images.

**(A)** a total of 25 radiomic features were identify by mRMR and LASSO logistic regression based on CT features. **(B)** list of 17 radiomic features chosen to construct CT radiomic model. **(C)** Representative results of CT radiomic model for predicting ALK rearrangement in training (left) and testing (right) group of lung adenocarcinoma patients. 0, negative ALK rearrangement; 1, positive ALK rearrangement.

**Supplementary Figure S2** Construction of a PET radiomic model based on PET images.

**(A)** a total of 14 radiomic features were identify by mRMR and LASSO logistic regression based on PET features. **(B)** list of 11 radiomic features chosen to construct PET radiomic model. **(C)** Representative results of PET radiomic model for predicting ALK rearrangement in training (left) and testing (right) group of lung adenocarcinoma patients. 0, negative ALK rearrangement; 1, positive ALK rearrangement.
