# Supplementary material for: A Machine Learning Model Based on PET/CT Radiomics and Clinical Characteristics Predicts ALK Rearrangement Status in Lung Adenocarcinoma
Source: Front Oncol. 2021 Mar 2;11:603882. doi: 10.3389/fonc.2021.603882 (PMC7962599; doi:10.3389/fonc.2021.603882)
Supplement: Supplementary file 5 [file Table_1.docx]

**Supplementary Table S1. The intra observer and inter observer ICCs of the consistency of lesions segmentation.**

|  | CT Images | PET Images |
| --- | --- | --- |
| Intra-group ICC | 0.38~1 | 0.45~1 |
| Remains(ICC>0.75) | 256 | 314 |
| Inter-group ICC | 0.39~1 | 0.44~1 |
| Remains(ICC>0.75) | 247 | 295 |
