# Supplementary material for: A Machine Learning Model Based on PET/CT Radiomics and Clinical Characteristics Predicts ALK Rearrangement Status in Lung Adenocarcinoma
Source: Front Oncol. 2021 Mar 2;11:603882. doi: 10.3389/fonc.2021.603882 (PMC7962599; doi:10.3389/fonc.2021.603882)
Supplement: Supplementary file 6 [file Table_2.docx]

**Supplementary Table S2. The final selected features which were used to construct PET/CT model.**

| **Parameter** |  |
| --- | --- |
| CT_uniformity | $\sum_{i=1}^{N_{g}} {p(i)}^{2}$, Uniformity measures the sum of the squares of each intensity value, which explains the homogeneity of the image array, the greater the uniformity implies a greater homogeneity. |
| CT_HaralickCorrelation_AllDirection_offset4_SD  CT_HaralickCorrelation_AllDirection_offset7_SD | $-\sum_{i,j} \frac{\left( i,j \right)^{g}\left( i,j \right)-u_{t}^{2}}{\sigma_{t}^{2}}$, HaralickCorrelation measures the degree of similarity of the grey level of the image in the row or column direction in different offset. Represents the local grey level correlation, the greater its value, the greater the correlation. |
| CT_ShortRunEmphasis_AllDirection_offset1_SD | $\frac{\sum_{i=1}^{N_{g}} \sum_{j=1}^{N_{r}} \frac{p(i,j\vert\theta)}{j^{2}}}{N_{r}(\theta)}$ , Short run Emphasis is a measure of the distribution of short run lengths, with a greater value indicative of shorter run lengths and more fine textural textures. |
| CT_HaraEntroy | $\sum_{i=1}^{N_{g}} \sum_{j=1}^{N_{g}} p\left( i,j \right)\log\left( p\left( i,j \right) \right)$. HaraEntroy is a measure of randomness, Quantifies the spatial gray-level co-occurrence matrix (GLCM) within local neighborhoods around each pixel in an image, stored in the form of matrices. |
| CT_LongRunLowGreyLevelEmphasis_AllDirection_offset4_SD  CT_LongRunLowGreyLevelEmphasis_AllDirection_offset7_SD  CT_LongRunLowGreyLevelEmphasis_AllDirection_offset1_SD | $\frac{\Sigma_{ⅈ=1}^{N_{g}}\sum_{j=1}^{N_{r}} \frac{P(i,j\vert\theta)}{i^{2}}}{N_{r}(\theta)},$ LongRunLowGreyLevelEmphasis measures the distribution of low gray-level values in all direction and offset, with a higher value indicating a greater concentration of low gray-level values in the image |
| PET_Percentile10  CT_Percentile70  CT_Percentile30 | P(a) = p%, Percentile in statistics indicating the value below which a given percentage of observations in a group of observations fall |
| CT_GLCMEnergy_angle135_offset7 | $\sum_{i,J} g\left( i,j \right)^{2}$This feature Returns the sum of squared elements in the GLCM in different angle and offset. The value is high when image has very good homogeneity or when pixels are very similar The Property Energy is also known as uniformity, uniformity of energy, and angular second moment |
| CT_GLCMEntropy_angle90_offset1 | $-\sum_{i,j} g\left( i,j \right)\log\left( i,j \right)$, Entropy is a measure of randomness of intensity image in the GLCM in different angle and offset, Entropy measures the loss of information or message in a transmitted signal and also measures the image information |
| CT_LongRunHighGreyLevelEmphasis_angle45_offset1  CT_LongRunHighGreyLevelEmphasis_angle90_offset4  CT_LongRunHighGreyLevelEmphasis_angle0_offset1 | $\frac{\Sigma_{ⅈ=1}^{N_{g}}\sum_{j=1}^{N_{r}} P(i,j\vert\theta)i^{2}}{N_{r}(\theta)}$, High Grey Level Emphasis measures lung length of the distribution of the higher gray-level values in different angle and different offset, with a higher value indicating a greater concentration of high gray-level values in the image. |
| CT_Correlation_AllDirection_offset4_SD | $-\sum_{i,j} \frac{\left( i-u \right)\left( j-u \right)g_{\left( i,j \right)}}{\sigma^{2}}$, Correlation measures the similarity of the grey levels in neighboring pixels, which shows how correlated a pixel is to its neighbor over the whole iamge. |
| CT_LongRunEmphasis_angle90_offset4  CT_LongRunEmphasis_AllDirection_offset1_SD  CT_LongRunEmphasis_angle135_offset4  CT_LongRunEmphasis_AllDirection_offset4_SD | $\frac{\Sigma_{ⅈ=1}^{N_{g}}\sum_{j=1}^{N_{r}} P(i,j\vert\theta)j^{2}}{N_{r}(\theta)}$, Long run emphasis measures the distribution of lung length in different angle and offset, the greater value is, the more coarse structural textures. |
| PET differenceEntropy | $\sum_{i=0}^{N\rho-1} P_{X-y}\left( i \right){log}_{2} \left( p_{x-y}\left( i \right) \right)$*,* Difference Entropy measures the randomness/variability in neighborhood intensity value differences. |

The offset means the pixels with different distance (1,4,7) of 1 pixel from each other.
