# Supplementary material for: A Machine Learning Model Based on PET/CT Radiomics and Clinical Characteristics Predicts ALK Rearrangement Status in Lung Adenocarcinoma
Source: Front Oncol. 2021 Mar 2;11:603882. doi: 10.3389/fonc.2021.603882 (PMC7962599; doi:10.3389/fonc.2021.603882)
Supplement: Supplementary file 7 [file Table_3.docx]

| **Supplementary Table S3. DeLong test of ROC curves from PET/CT、CT、PET models.** | | |
| --- | --- | --- |
| **Comparisons** | **Z score** | **p value** |
| PET/CT radiomic model vs. CT radiomic model | 0.327 | 0.744 |
| PET/CT radiomic model vs. PET radiomic model | 0.408 | 0.684 |
| CT radiomic model vs. PET radiomic model | 0.118 | 0.906 |
