# Supplementary material for: A Machine Learning Model Based on PET/CT Radiomics and Clinical Characteristics Predicts ALK Rearrangement Status in Lung Adenocarcinoma
Source: Front Oncol. 2021 Mar 2;11:603882. doi: 10.3389/fonc.2021.603882 (PMC7962599; doi:10.3389/fonc.2021.603882)
Supplement: Supplementary file 8 [file Table_4.doc]

| **Supplementary Table S4. Univariate logistic analysis of clinical features and ALK mutation status.** | | |
| --- | --- | --- |
| **Variables** | **OR (95% CI)** | **p value** |
| **Age** | 0.93（0.91-0.96） | <0.001 |
| **Burr** | 0.34（0.13-0.90） | 0.025 |
| **Pleural adhesion** | 1.81（1.09-3.02） | 0.021 |
| **Maximum length** | 1.30（1.08-1.56） | 0.006 |
| **Pleural effussion** | 64.67（12.65-1182.60） | <0.001 |
| **Ground glass** | 0.19（0.09-0.36） | <0.001 |
| **Calcification** | 3.15（0.76-12.17） | 0.094 |
| **Stages** | 2.13（1.69-2.73） | <0.001 |
