# Supplementary material for: A Machine Learning Model Based on PET/CT Radiomics and Clinical Characteristics Predicts ALK Rearrangement Status in Lung Adenocarcinoma
Source: Front Oncol. 2021 Mar 2;11:603882. doi: 10.3389/fonc.2021.603882 (PMC7962599; doi:10.3389/fonc.2021.603882)
Supplement: Supplementary file 9 [file Table_5.doc]

| **Supplementary Table S5. Multivariate logistic analysis of clinical and radiomic features and ALK mutation status.** | | |
| --- | --- | --- |
| **Variables** | **OR (95% CI)** | **p value** |
| **Age** | 0.93（0.90-0.96） | <0.001 |
| **Burr** | 0.39（0.12-1.39） | 0.129 |
| **Pleural effussion** | 16.92（2.70-332.09） | 0.011 |
| **Radscore** | 1.97（1.61-2.45） | <0.001 |
| **Intercept** | 46.83（5.61-418.49） | <0.001 |
