# Supplementary material for: A Machine Learning Model Based on PET/CT Radiomics and Clinical Characteristics Predicts ALK Rearrangement Status in Lung Adenocarcinoma
Source: Front Oncol. 2021 Mar 2;11:603882. doi: 10.3389/fonc.2021.603882 (PMC7962599; doi:10.3389/fonc.2021.603882)
Supplement: Supplementary file 10 [file Table_6.doc]

| **Supplementary Table S6. DeLong test of ROC curves from Integrated, PET/CT radiomic and Clinical models.** | | |
| --- | --- | --- |
| **Comparisons** | **Z score** | **p value** |
| Integrated model vs. PET/CT radiomic model | 0.534 | 0.593 |
| Integrated model vs. Clinical features model | 4.353 | <0.001 |
| PET/CT radiomic model vs. Clinical features model | 2.271 | 0.023 |
